# The Possible Pathogenesis of Idiopathic Pulmonary Fibrosis considering* MUC5B*

**DOI:** 10.1155/2019/9712464

**Published:** 2019-06-11

**Authors:** Qinghua Zhang, Yan Wang, Danhua Qu, Jinyan Yu, Junling Yang

**Affiliations:** Department of Respiratory Medicine, The Second Hospital of Jilin University, Changchun, Jilin Province 130041, China

## Abstract

**Background:**

Overexpression of the MUC5B protein is associated with idiopathic pulmonary fibrosis (IPF), but little information is available regarding the pathogenic effects and regulatory mechanisms of overexpressed MUC5B in IPF.

**Main Body:**

The overexpression of MUC5B in terminal bronchi and honeycomb cysts produces mucosal host defensive dysfunction in the distal airway which may play an important role in the development of IPF. This review addresses the possible association of overexpression of* MU*C5B, with* MUC5B* promoter polymorphism,* MUC5B* gene epigenetic changes, effects of some transcriptional factors, and inflammatory mediators in IPF. In addition, the associated signaling pathways which may influence the expression of* MUC5B* are also discussed.

**Conclusion:**

This work has important implications for further exploration of the mechanisms of overexpression of MUC5B in IPF, and future personalized treatment.

## 1. Introduction

Idiopathic pulmonary fibrosis (IPF), a lung disease of unknown etiology, is characterized by progressive lung scarring of the pulmonary parenchyma and the formation of subpleural honeycomb cysts. The published incidence of IPF has ranged from 0.6 per 100,000 person years to 17.4 per 100,000 person years in different areas from 1981 to 2009 [1, 2]. IPF is characterized by progressive dyspnea, respiratory failure, and overall poor prognosis, and the median term of survival after diagnosis is only about 3–5 years [3]. However, there is prolonged survival (6–12 yrs) in up to 20% of IPF patients [4]. Over the last decade, the treatment of IPF has been revolutionized by substantial advances in understanding of the pathogenic mechanisms, which has changed from a postulated uncontrolled inflammation, for which there is a lack of adequate support, to alveolar epithelial cell dysfunction, immune dysregulation, fibroproliferation/fibrogenesis/matrix remodeling, and aberrant regeneration of airway epithelia [4–6].

IPF has a known genetic component, as evidenced by the fact that as many as 1/5 of affected individuals report having a family member with pulmonary fibrosis [7]. Genetic susceptibility to familial interstitial pneumonia (FIP) and sporadic IPF is probably related to several gene variants that result in alveolar epithelial cell dysfunction [8]. In these variants, telomerase reverse transcriptase (*TERT*), telomerase RNA component (*TERC*), poly(A)-specific ribonuclease (*PARN*), and regulator of telomere length elongation helicase (*RTEL*) are implicated in the maintenance of telomere length, and surfactant protein C (*SPC*) and surfactant protein A2 (*SPA2*) have been recognized to maintain surfactant function [9–11]. Besides, Noth et al. [12] have identified other genetic variants as risk factors of IPF, including mucin 5B (*MUC5B*), desmoplakin (*DSP)*, phospholipid transporting 11A ATPase, and toll interacting protein (*TOLLIP*), which play important roles in the maintenance of bronchoalveolar epithelium function and immune regulation [8].

Up to now, extensive genome-wide linkage scanning has identified a single nucleotide polymorphism (SNP) in the promoter region of the* MUC5B* gene (rs35705950) that is the principal risk factor (genetic and otherwise) for developing IPF, accounting for 30–35% of the risk [4, 12–19].

MUC5B protein may play a direct role in the pathogenesis of IPF regardless of the allele status [19]. The identification of MUC5B as a common risk factor has altered our view of the pathogenesis of pulmonary fibrosis from alveolar epithelial cell injury and matrix deposition to mucus overproduction in the distal airways. Many investigators posit the theory that persistent bronchiolar epithelial injury and the overproduction of MUC5B by airway progenitor cells result in the development of honeycomb cysts and IPF [4, 19, 20]. Therefore, elucidating the mechanistic role of MUC5B in IPF could lead to important advances in our understanding of the pathogenesis of FIP and sporadic IPF. In this review, we summarize the current knowledge of the production and regulation of MUC5B, including the potential mechanism of MUC5B in the occurrence and development of IPF.

## 2. MUC5B Expression in IPF

### 2.1. Mucins in Normal Airways

In the normal lungs, mucus is responsible for trapping inhaled particles, including bacteria, and transporting them out of the airways by ciliary and cough-driven forces. Meanwhile, mucus also helps remove endogenous debris including dying epithelial cells and leukocytes [21, 22]. Mucins, the glycosylated proteins in mucus, are primarily responsible for giving mucus their viscoelastic properties. Up to now, about 20 mucin genes have been identified. Among these, 11 are expressed in the lungs, including MUC1, 2, 3, 4, 5AC, 5B, 6, 7, 8, 13, and 19 [23]. Among these, MUC2, MUC5AC, MUC5B, and MUC19 are secreted, and MUC5AC and MUC5B are the major mucins.

MUC5AC and MUC5B are expressed throughout the upper and lower respiratory tract. In the tracheobronchial conducting airways, both mucins are synthesized by surface goblet cells and submucosal glandular cells. It has been confirmed in Seibold et al.'s study [20] that the proximal airway (airways that are supported by cartilage and contain submucosal glands) epithelium is populated by cells expressing MUC5AC and coexpressed MUC5B, except for the submucosal glands populated by mucus cells expressing MUC5B and little MUC5AC. In distal airways (airways that lack cartilage and submucosal glands), MUC5B is the dominant gel-forming mucin, and little MUC5AC is present. Distal airways that express MUC5AC are a subset of the MUC5B+ distal airways, and distal airways that express only MUC5AC are not detected [4, 20, 21, 24].

### 2.2. Expression of MUC5B in IPF

IPF has always been considered to primarily involve the alveolar region [25]. However, several lines of evidence suggest that aberrant distal airway processes may also contribute to IPF pathogenesis.

Except for the aberrant physiologic alterations (e.g., forced expiratory volume in 1s/forced vital capacity, maximum expiratory flow-volume curves, and maximum flow static recoil curves) which are consistent with distal airways abnormalities in IPF [26], earlier histological studies have also suggested that distal airways are involved in interstitial lung diseases [26, 27]. A recent histological study [28] shows that patients with usual interstitial pneumonitis (UIP) present increased bronchiolar inflammation and peribronchiolar inflammation as well as fibrosis and have thicker bronchial walls compared to the control. Moreover, novel information related to the increased expression of TGF-*β*, MMP-9, and MMP-7 in the bronchiolar epithelium in UIP patients is also provided by this study. In the perspective of imaging features, it is suggested that, in the earliest stages of IPF development, patients have the increased measures of an internal perimeter of 10mm (Pi10) which are commonly viewed as increased measures of airway wall thickness [29]. Taken together, these data pointed to the possibility that distal airway involvement occurred as part of IPF pathogenesis.

Besides the histological studies, a growing body of evidence from genetics has implicated biologic processes occurring in the distal airways as potential drivers of IPF pathogenesis. For example, the promoter SNP rs35705950 in* MUC5B* gene, the intron 5 variant rs2076295 in desmoplakin (*DSP) *gene, and their enhanced expression localized to the distal airway epithelial cells are considered associated with IPF pathogenesis [20, 30–32]. In the present study, we focus on the analysis of MUC5B in IPF providing valuable insights into the role of distal airways in IPF pathogenesis.

In Seibold et al.'s study [20], dual immunofluorescence analysis of MUC5B and MUC5AC demonstrates that the distal airways are populated by mucus cells expressing MUC5B in 80% of IPF subjects. Up to 75% of these distal airways also contain epithelial cells expressing MUC5AC. The frequencies of MUC5B^+^ / MUC5AC^+^ distal airways in IPF patients are both significantly greater than those in normal subjects, and the MUC5B^+^ distal airways are especially more frequent. Perhaps the conversion of MUC5B^−^ distal airways to MUC5B^+^ airways and the increase of cells expressing MUC5B in distal airways occur in IPF subjects.

It is well established that the characteristic changes of HRCT in IPF/UIP subjects are always subpleural honeycomb cysts and lower zone predominant reticular infiltrates. However, there exists another form of honeycomb cysts in IPF, namely, microscopic honeycomb, which does not correlate with the honeycomb observed in HRCT representing dilated airways due to traction from the fibrotic process. Epithelial cells expressing MUC5B are the dominant mucin-expressing cell type in microscopic honeycomb cysts. The microscopic honeycomb cysts are filled with MUC5B protein and chronic inflammation cells and lined by pseudostratified ciliated columnar epithelium similar to those which line the bronchioles [19, 20, 33, 34]. MUC5B immunohistochemical staining has shown a dense accumulation of MUC5B in microscopic honeycomb cysts as well as pseudostratified bronchial epithelium and terminal bronchioles in Seibold et al.'s another study [19]. In addition, accumulating evidence suggests that the distal airway epithelium which is the principal origin of alveolar stem cells expressing Krt5 could give rise to type I and type II alveolar cells and migrate to occupy the injured areas after bleomycin challenge and H1N1 infection in mice [35–37]. The aberrant differentiation of distal airway progenitors expressing Krt5 is in close association with MUC5B rich honeycomb cysts [20]. Therefore, these investigators posit that the MUC5B rich microscopic honeycomb cysts are derived from the bronchioles and place emphasis on the role of distal airways in IPF pathogenesis. This hypothesis is disputable, since it has been long thought that microscopic honeycomb cysts derive from type II alveolar epithelial cells and represent the reconstruction of alveolar structures in the end stages of IPF/UIP [38–40].

### 2.3. Pathological Effects of MUC5B in IPF

Mucus overproduction contributes to the morbidity of many airway diseases, among which the most common are chronic obstructive pulmonary disease (COPD), asthma, cystic fibrosis (CF), and diffuse chronic panbronchiolitis [41–43]. In addition, many researchers have speculated that the overproduction of MUC5B contributes to the development of IPF resulting from excessive lung injury and aberrant repair [19, 44–46]. Although the mechanisms remain unclear, the associated hypotheses or possibilities are consistent [16, 19, 46, 47].

First, excessive MUC5B compromises the mucosal host defense and reduces lung clearance of inhaled particles, dissolved chemicals, and microorganisms. Over time, reduced clearance may lead to scar tissue formation and persistent fibroproliferation that expands and displaces normal lung tissue. Given that cigarette smoking is a strong risk factor for the development of IPF [48], it is logical to speculate that the inhaled particles associated with cigarette smoking might cause defects in mucosal host defense and subsequent interstitial injury.

Second, excessive MUC5B in the respiratory bronchioles may interfere with alveolar repair. It has been established that local expansion of type II alveolar epithelial cells following lung injury may repopulate denuded alveolar basement membranes [49, 50]. One possibility is that MUC5B impedes alveolar repair either by interfering with the interaction between type II alveolar epithelial cells and the underlying matrix or by interfering with the surface-tension properties of the surfactant [19]. The failure to reepithelialize damaged alveoli could enhance the collapse and fibrosis of bronchoalveolar units and eventually lead to the development of idiopathic pulmonary fibrosis.

The changes of MUC5B in the distal conducting airways potentially enhance injury or disrupt repair responses in alveoli. The two mechanisms are plausible and may act alone or together to contribute to the development of idiopathic pulmonary fibrosis.

## 3. Influence of* MUC5B* Expression

The overproduction of MUC5B plays an important role in the development of IPF; however, little is known about the influencing factors of gene expression. Identifying the associated influencing factors of the* MUC5B* gene in pulmonary diseases will contribute to the exploration of explicit signaling pathways in MUC5B overproduction. At present, genetic mutation, epigenetic changes, effects of some transcriptional factors as well as inflammatory mediators, and the associated signaling pathways may be involved in the regulation of MUC5B production.

### 3.1. Methylation of CpG Motifs in a MUC5B Promoter Variant Increases the Expression of MUC5B

From allelic testing, Seibold et al. [19] observed the significant associations of 19 independent SNPs with familial interstitial pneumonia, idiopathic pulmonary fibrosis, or both. Of these 19 SNPs, five are in the region of the* MUC5B* promoter, within 4 kb of the* MUC5B* transcription start site. An SNP, rs35705950, is a G to T transversion that occurs in an area of the* MUC5B* 5'flanking region, 3 kb upstream of the transcription start site, exhibited the strongest association with both familial interstitial pneumonia and idiopathic pulmonary fibrosis. In a survey conducted in a European Caucasian population, the T risk allele was present in 41.9% of the IPF patients and 10.8% of the controls [16], while in a Chinese population, the frequency of the T allele was approximately 3.33% in IPF patients and 0.66% in controls [51]. Although the minor-allele frequency of the polymorphism rs35705950 in Chinese populations is lower than that in Caucasian populations, the variant in* MUC5B *promoter is still significantly associated with the risk for IPF.

Although the polymorphism in the promoter of* MUC5B* is strongly associated with the development of IPF, there are still many individuals with this SNP rs35705950 that do not develop to IPF [13, 19]. Therefore, even sharing the same genetic variant, different individuals do not necessarily have the same disease pattern. In this situation, other influences such as environmental factors are pivotal to the development of IPF. Up to now, the well-known environmental factors associated with IPF include occupational exposure such as asbestos [52], cigarette smoking [53], and some viruses [54] such as hepatitis C, adenovirus, and herpesvirus. These environmental factors may influence the epigenetic changes of* MUC5B* gene, thus changing the expression of* MUC5B. *Epigenetic changes, such as CpG motif methylation, belong to the non-Mendelian genetics, but are heritable and always influenced by the environmental factors [55]. Therefore, we speculate the epigenetic changes in* MUC5B* promoter may establish a link between environmental exposures and the* MUC5B* promoter polymorphism in IPF.

Many studies show that the* MUC5B* promoter variant rs35705950 has been validated as a contributor to the expression of MUC5B in the lung [14–16, 45, 51, 56, 57]. Efforts are underway to understand how this polymorphism in the* MUC5B* promoter contributes to the expression of* MUC5B* in IPF. This polymorphism may be related to the methylation in the* MUC5B* promoter region and the disruption of the activities of some transcriptional factors.

Previous epigenetic analysis of the* MUC5B* promoter region showed that* MUC5B* was highly sensitive to DNA methylation [58]. Helling et al. [59] further evaluated the association between the variant, rs35705950, and DNA methylation. They found that a differentially methylated region containing 11 CpG motifs (Chr11: 1241139–1241412) is associated with the presence of the* MUC5B* promoter variant (Chr11: 1240989–1241950),* MUC5B* expression, and IPF. This region is hypermethylated in association with IPF, increased expression of* MUC5B* in lung tissue, and the rs35705950 risk allele (T). These findings define a regulatory region and support a role for DNA hypermethylation in the regulation of* MUC5B* expression in the lung and as a risk factor for the development of IPF.

### 3.2. Effects of Other Mutations on MUC5B Expression

As previously mentioned, mutations in the genes for* SPC* and* SPA2 *have been described in association with FIP and rarely with sporadic IPF. Considering that the* MUC5B* promoter variant rs35705950 is associated with adult IPF, Liptzin et al. [60] examined whether MUC5B is similarly linked to pediatric IPF secondary to* SPC* mutations. The study showed that MUC5B was increased in BALF and lung tissues of pediatric IPF patients with* SPC* mutations compared with the controls, indicating that MUC5B may play a role in the development of IPF in patients with* SPC* mutations. Previous studies suggested that the mutations in* SPC* could lead to endoplasmic reticulum (ER) stress [61–63] which may account for the MUC5B overproduction shown in Liptzin's study and indicates a need for further study.

Besides* MUC5B* rs35705950, genome-wide association studies (GWAS) have identified other polymorphisms conferring risks to IPF. Among these loci, rs2736100 in* TERT *genes were also identified in several independent studies [15, 64]. Wei et al. [17] tested the associations between rs2736100 and rs35705950 in IPF cases and controls; however no significant deviation was found, suggesting that the* TERT* and* MUC5B* polymorphisms independently and differentially confer susceptibility to IPF.

TOLLIP, a regulator of innate immune responses involved in modulating Toll-like, receptor signaling resides at the same genetic locus with the* MUC5B *gene. The study conducted by Noth et al. [12] showed the variants of* TOLLIP* and* MUC5B* are in weak linkage disequilibrium, and the association of* TOLLIP* genetic variants with IPF susceptibility is independent from that of the* MUC5B* promoter SNP. However, whether the* MUC5B* promoter variant rs35705950 is a pathogenic determinant independent of other mutations remains to be elucidated.

### 3.3. Regulation of MUC5B by Transcription Factors

As mentioned above, the increased methylation in MUC5B promoter region may directly affect MUC5B expression. Conversely, methylation will also affect the binding of some transcription factors to the* MUC5B *gene regulatory region.

**Figure 1:**
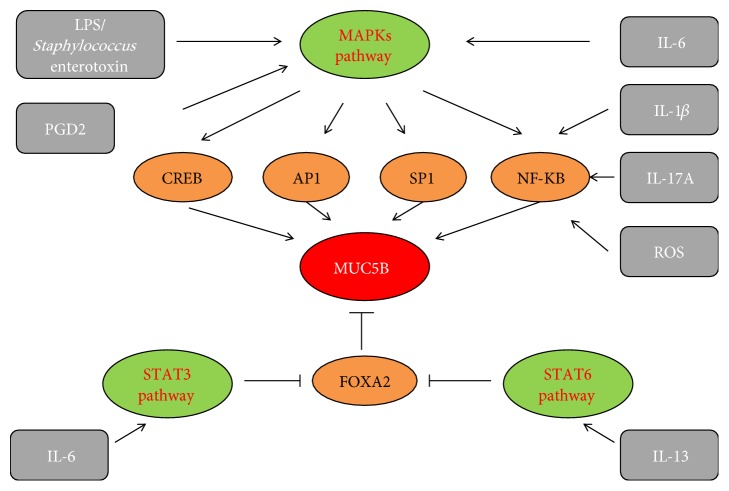
*Regulation of MUC5B expression in airway epithelial cells.* Different stimuli, including some interleukins, ROS, PGD2, and certain bacterial components may induce MUC5B overexpression by MAPKs, STAT3, or STAT6 pathways. The important transcriptional factors including FOXA2, CREB, AP-1, SP1, and NF-*κ*B also directly or indirectly participate in* MUC5B* overexpression.

To date, in the 5' region upstream of the* MUC5B* gene transcriptional start site, numerous several transcriptional factor binding sites have also been identified, including the motifs binding forkhead box A2 (FOXA2), activator protein 1 transcription factor (AP-1), Sp1, NF-*κ*B, TTF-1, TGT3, CREB, STAT, c-Myc, and others [65, 66]. These motifs may become active after binding to the corresponding transcriptional factors in the induction of* MUC5B *gene expression (Figure 1). However, at present, only a few transcriptional factors have been tested experimentally in the context of their involvement in* MUC5B *expression regulation in a variety of diseases including cancer and lung fibrosis [65, 67]. 

*FOXA2 Mediated Transcriptional Regulation of MUC5B.* According to a study by Helling et al. [59], forkhead box A2 (FOXA2), one of the transcription factors residing in the region 32 bp downstream of rs35705950, is highly conserved across mammals ranging from primates to rodents and has the most significant effects on* MUC5B* expression. The* MUC5B* promoter variant rs35705950 elicits methylated changes surrounding the FOXA2 binding motif, and more occupancy of FOXA2 in the binding motif leads to an increase of* MUC5B* expression [59]. Another study by Hao et al. [68] regarding the modification of FOXA2 by pyocyanin demonstrated that pyocyanin might cause posttranslational modifications (nitrosylation, acetylation, and ubiquitination) of FOXA2. The modified FOXA2 is degraded and has reduced ability to bind the promoter of the* MUC5B* gene. The degradation and functional impairment of FOXA2 are positively correlated to the elevation of MUC5B biosynthesis instead. The contrary effects of FOXA2 on* MUC5B* expression reinforce that the regulation of* MUC5B* is complex, and perhaps other factors regulate* MUC5B* expression independent of promoter polymorphism and FOXA2. 

*Sp1 Mediated Transcriptional Regulation of MUC5B.* Sp1 is a member of the multigene family that binds DNA through COOH-terminal zinc-finger motifs [69] and can be phosphorylated under various circumstances by a variety of kinases. Phosphorylated Sp1 may regulate the expression of several genes that are relevant to human pathologic condition, including vascular endothelial growth factor (*VEGF*) [70], type I collagen (*COL1A2*) [71], transforming growth factor beta* TGF-β *[72], matrix metalloproteinases (*MMPs*) [73], and* MUC5B *[67].

In order to identify the regulatory regions involved in* MUC5B *transcription, the* MUC5B* promoter region in three intestinal cancer cell lines has been sequenced and analyzed by Van Seuningen and collaborators [65]. They reveal that* MUC5B* promoter contains a high number of GC and CACCC boxes; those are the motifs binding Sp1. In human airway epithelial cells, Chang's group [74] used chromatin immunoprecipitation (ChIP) assays to confirm that Sp1 also participates in the regulation of* MUC5B *via binding to* cis-*Sp1 sites in* MUC5B* promoter. The further research by Wu et al. [74] has shown that the phosphorylation or the activation of Sp1 can increase its DNA-binding activity to the* MUC5B* promoter, thereby promoting* MUC5B* expression. At present, although there is a limit data about the regulation of* MUC5B* by Sp1 in IPF, Kum et al. [67] have proved blocking the activity of Sp1 at DNA level is an effective approach on lung fibrosis treatment. Therefore, we speculate Sp1 plays a role in promoting* MUC5B* expression in IPF. 

*NF-kB Mediated Activation of MUC5B.* According to the study by Van Seuningen et al. [65], Sp1 binding motifs in* MUC5B* promoter are found to be nearby the binding motifs for other transcriptional factors. It indicates that the clustered transcriptional factors including Sp1 may act together to modulate transcription of* MUC5B* [75]. Among the other factors, NF-*κ*B also participates in the regulation of* MUC5B* transcription.* MUC5B *promoter region contains several putative NF-*κ*B binding sites in murine middle ear epithelial cells, and there are two possible NF-*κ*B* cis-*sites in the −556 bp* MUC5B* promoter region and one NF-*κ*B* cis-*site in the −229 bp region [76]. Studies have shown that cigarette smoking, associated with IPF genesis in patients of different ethnic backgrounds [77], can activate many genes with NF-*κ*B* cis*-sites in their 5' upstream flanking sequences in human lung epithelial cells [78, 79]. Although there is a lack of sufficient evidence about the activation of NF-*κ*B and induction of* MUC5B* in distal airway epithelium, however, the study [76] has demonstrated both qualitatively and quantitatively that cigarette smoking upregulates MUC5B mRNA levels partly mediated by activated NF-*κ*B in murine middle ear epithelial cells. With regard to the upstream signaling molecules of NF-*κ*B activation, the phosphorylation of P38 MAPK has been confirmed by quite a few studies [76, 80–82]. 

*AP-1 Mediated Regulation of MUC5B.* AP-1, a dimeric complex of related transcriptional factors containing members of the JUN, FOS, ATF, and MAF protein families, regulates a wide range of cellular processes including cell proliferation, apoptosis, migration, and differentiation. Previous studies suggest that there are intersections between the NF-*κ*B pathway and AP-1 pathways, and many stimuli activating NF-*κ*B will also activate AP-1 [83, 84] whose upstream is P38 MAPK [85]. The activated AP-1 could promote the production of proinflammatory mediators and profibrotic factors in pulmonary fibrosis. Li et al. [86] have proved that gefitinib could relieve pulmonary fibrosis by inhibiting the activation of NF-*κ*B, c-Jun, and c-Fos. Considering the association between AP-1 and MUC5B, Lee et al. [87] have provided a series of proof for the inhibition of respiratory syncytial virus inducing MUC5B synthesis in human airway epithelial cells, which is related to the downregulation of p38 MAPK/AP-1 pathway. Similarly, Li et al. [88] proved the hypersecretion of airway mucus including MUC5A and MUC5B in pneumonia induced by respiratory syncytial virus is related to the activation of AP-1. Therefore, the activated MAPKs and downstream AP-1 as well as NF-*κ*B are believed to be associated with the overexpression of MUC5B and lung injury in IPF in our study. 

*CREB Mediated Regulation of MUC5B.* cAMP-response element-binding protein (CREB) could recognize the specific DNA sequences 5'- TGACGTCA- 3', which is known as cAMP response element (CRE) in the transcription regulatory region of many genes [89, 90]. The involved upstream kinases in response to various stimuli in the activation of CREB include protein kinase A (PKA) [91], protein kinase C [92], and p90 ribosomal S6 kinase 1/2 (RSK1/2) [93] as well as mitogen-activated protein kinase (MAPK) [94]. The phosphorylation of CREB binds to the specific* cis-*CRE sites (-1011, -1032, -1165, -1252) in the* MUC5B* promoter to upregulate* MUC5B* expression in human primary airway epithelial cells [94]. Previous study [95] indicated that the phosphorylation may allow the recruitment of a coactivator, CREB binding protein (CBP), which augments the activity of phosphorylated CREB to activate transcription of cAMP-responsive genes, while the inhibition of CBP has been confirmed to reverse pulmonary fibrosis [96].

The above studies indicate the important role of the transcriptional factors SP1, AP-1, NF-*κ*B, and CREB and activated MAPKs signaling in the overexpression of* MUC5B* in pulmonary fibrosis. However, the regulation of* MUC5B* is considered a cell-specific process, and the special roles of the transcriptional factors in* MUC5B* expression in distal airway epithelium in IPF patients have not been characterized. It deserves further study, and we believe the greater understanding of MUC5B regulation by transcription factors may provide a new perspective on the treatment of IPF.

### 3.4. Regulation of MUC5B by Inflammatory Mediators

Although the role of inflammation in IPF is controversial, inflammatory mediators are indeed critical factors in the pathogenesis of IPF [97]. Meanwhile, several classes of inflammatory mediators have also been implicated in the process of MUC5B hypersecretion. These inflammatory mediators mainly include cytokines, bacterial components, reactive oxygen species (ROS), and arachidonic acid metabolites (Figure 1) [80, 98–103].

#### 3.4.1. Cytokines Mediated Regulation of MUC5B

Over the last decades, considerable evidence has suggested that cytokines play important role of stimuli in pulmonary fibrosis development [104]. Released by resident lung cells, such as macrophages, epithelial and endothelial cells, cytokines are thought to stimulate fibroblast proliferation and increase synthesis of extracellular matrix. In addition, cytokines are also proved to regulate mucin production in airway epithelial cells [98, 105]. Related studies reveal that a large number of cytokines, including IL-1*β*, IL-4, IL-6, IL-9, IL-10, IL-13, IL-17, TNF-*α*, and IFN-*γ*, are known to regulate mucin synthesis [98, 106, 107]. Of these cytokines, IL-1*β*, IL-6, IL-13, and IL-17A have been proved to upregulate MUC5B in airway epithelial cells in many studies [98–101].

In pulmonary fibrosis, IL-1*β* is found to be upregulated [108], and the overexpression of IL-1*β* in rat lungs promotes the presence of myofibroblasts, fibroblast foci, and extracellular matrix accumulation which are the characteristics of pulmonary fibrosis [109]. IL-1*β* is also elevated in lungs of patients with MUC5B hypersecretion respiratory disease, such as chronic obstructive pulmonary disease (COPD) and cystic fibrosis (CF) [41, 110–112]. The IL-1 receptor type 1 (IL1R1), a common receptor for IL-1, always mediates the downstream signaling pathway in multiple types of cells. Chen et al. [105] tested the effects of IL-1 in the CF lung on MUC5B expression in primary human bronchial epithelial cells and proved that IL-1R1 mediated IL-1 induced* MUC5B *overexpression. Further study suggested the binding of IL-1*β* and IL-1R1 is followed by NF-*κ*B-based transcriptional mechanism [81]. Therefore, it is easy to associate IL-1*β* induced MUC5B overexpression in airway epithelium participates the development of IPF.

IL-17A, produced by CD4+ and *γδ*+ T cells, has been confirmed to induce significant neutrophilia and pulmonary fibrosis after exposure to bleomycin [113–115]. Bleomycin induced IL-17A production is TGF-*β* dependent, suggesting the cooperative roles for IL-17A and TGF-*β* in the development of fibrosis [113]. In addition, the study [13] also showed that IL-17A and IL-1*β* are both increased in the bronchoalveolar lavage fluid of patients with IPF, and the fibrogenic effect by IL-1*β* is also dependent on IL-17A. Therefore, the increased IL-17A also plays a critical role in pulmonary fibrosis. Fujisawa et al. [81] show that IL-1*β* and IL-17A could active* MUC5B* promoter resides within the -4.17kb to -2.56kb region relative to* MUC5B* promoter transcriptional start site, containing putative NF-*κ*B binding sites. The activated NF-*κ*B based transcriptional mechanism by IL-17A is involved in MUC5B regulation in human bronchial epithelial cells [81, 116].

IL-13 typically serves a protective role and has been implicated as a key regulator of epithelial cell biology. However, disregulated IL-13 will exert powerful profibrogenic effects in numerous chronic fibrotic diseases, including interstitial lung disease [99, 117, 118]. An initial stimulus, such as* M. pneumoniae*, viruses, or cigarette smoking will lead to the production of IL-13 which is a critical driver for mucus production [100, 119, 120]. Previous study suggested that IL-13 and IL-4 could share the common receptor IL-4R*α*, thus activated transcription 6 (STAT6) signaling pathway in airway epithelial cells [121], which in turn downregulate FOXA2, a transcriptional repressor of MUC5B biosynthesis [100, 101, 122]. Similarly, in another study, the expression of FOXA2 was confirmed to be decreased or absent in airway epithelial cells transgenic mice overexpressing IL-13, also suggesting that IL-13 could downregulate FOXA2 [123].

The cytokine IL-6 functions as a proinflammatory factor as well as a profibrotic factor in pulmonary fibrosis [124, 125], and the signaling pathway of IL-6/Stat3 has been shown to play an important role in the pathogenesis of lung fibrosis [126]. In addition, the study showed that IL-6 was also able to upregulate mucin secretion partly through the activation of Stat3 [127]. The stimulation of* MUC5B* expression by IL-6 has been proved in human tracheobronchial epithelial cells in Chen's study [98], and they have further demonstrated that IL-6 mediates* MUC5B* expression through the ERK signaling pathway.

Taken together, the studies indicate the important role of cytokines above and their related downstream signaling molecules in the overexpression of* MUC5B* in pulmonary fibrosis.

#### 3.4.2. Bacterial Components Induce MUC5B Production

A number of bacterial components have been implicated in mucin gene regulation [106, 128]. Lipopolysaccharide (LPS) and* Staphylococcus* enterotoxin are the most representative bacterial components that can induce* MUC5B* production.

LPS is located on the outer membrane of gram-negative bacteria and recognized by LPS-binding protein (LBP), CD14, and Toll-like receptor 4 (TLR4) leading to a robust proinflammatory response in mammalian cells including macrophages, epithelial cells, and fibroblasts [129]. A lot of studies have shown that LPS plays an important role in the development of acute lung injury, acute respiratory distress syndrome. In addition, LPS could also induce pulmonary fibrosis, mainly through activation of TLR4 and its downstream intracellular signal transduction pathways [130]. Several studies have linked LPS to the induction of* MUC5B* expression in human NCI-H292 airway epithelial cells [102, 131]. Bae et al. [102] have confirmed that the promotion of* MUC5B* by LPS induces TLR4 expression and the phosphorylation of ERK1/2 and p38 MAPK. Conversely, TLR4 siRNA-mediated knockdown of TLR4 significantly blocked LPS-induced* MUC5B* expression. These findings suggest that TLR4, ERK1/2 MAPK, and p38 MAPK play essential roles in LPS-induced* MUC5B *expression in human airway epithelial cells.

Similar to LPS,* Staphylococcus aureus* enterotoxins are known to induce an inflammatory airway response. Although there is little information on the possible role of* Staphylococcus aureus enterotoxins* in the development of pulmonary fibrosis, however, previous animal study has demonstrated that the intratracheal administration of* Staphylococcus aureus* enterotoxins A and E could induce development of interstitial pneumonia [132]. In the recent study conducted by Kim et al. [133], they reported that* Staphylococcus aureus* enterotoxin B could induce endoplasmic reticulum stress via reactive oxygen species production in airway epithelial cells. Endoplasmic reticulum stress plays an important role in the regulation of* MUC5B* via the activations of X-box binding protein 1 (XBP-1), activating transcription factor 6 (ATF6), and CCAAT-enhancer-binding protein homologous protein (CHOP) in human airway epithelial cells [134]. In addition, Song et al. [128] showed that* Staphylococcus aureus* enterotoxin A induced* MUC5B *expression by significantly inducing Toll-like receptor 2 (TLR2) expression and activating phosphorylation of ERK1/2 and p38 MAPK in human airway epithelial cells.

#### 3.4.3. ROS Induce* MUC5B* Production

ROS generated from oxidative stress are implicated as important molecular mediators in the development of fibrosis in a variety of organs including the lungs. However, the causal role of ROS in mediating MUC5B overproduction has not been firmly established.

ROS produced by cytokines, growth factors, and vasoactive agents contribute to the intracellular signaling cascades associated with inflammatory responses, including the ROS-dependent NF-*κ*B signaling pathway, for example [135]. A study conducted by Song [80] showed visfatin, an immunomodulator inducing upregulation of the pro- and anti-inflammatory cytokines in human monocytes, may significantly increase* MUC5B* expression via ROS formation and the NF-*κ*B signaling pathway in human airway epithelial cells.

Alternately, higher levels of ROS may trigger DNA damage, p53 activation, cell cycle blockade, and apoptosis. It is well known that the most common gene mutations identified in familial pulmonary fibrosis patients and some sporadic IPF patients are in the telomerase genes* TERT *and* TERC* [136]. These mutations and shortened telomeres are in part due to ROS, including mitochondrial-derived ROS in senescence [137, 138]. Considering the polymorphism in the* MUC5B* promoter region in some patients with sporadic IPF and familial pulmonary fibrosis, further studies are necessary to determine how ROS adversely impacts the* MUC5B *gene and its protein product in the development of pulmonary fibrosis in humans.

#### 3.4.4. Prostaglandin D2 (PGD2) Induces MUC5B Production

Prostaglandin (PG) 2 is a potent biologically active lipid mediator that is produced from arachidonic acid by almost every type of cell [139]. One of them, prostaglandin D2 (PGD2), is thought to be involved in inflammation [140]. In consideration of the effects of inflammatory mediators to* MUC5B* gene expression, Choi et al. [103] investigated the mechanisms by which PGD2 induces* MUC5B* gene expression in airway epithelial cells. The study suggests that PGD2 acts as a key factor of* MUC5B* gene expression via the D-prostanoid receptor (DP1) in human airway epithelial cells. PGD2 binding to DP1 induced the ERK MAPK/RSK1/CREB activation. The p90 ribosomal S6 kinases (RSK) are a family of serine/threonine kinases that are activated downstream of the Ras/MAPK pathway. RSK phosphorylates multiple signaling effectors to play an essential role in numerous cellular functions, including regulation of gene expression through the phosphorylation of transcriptional regulators such as CREB. RSK1 is a mediator of CREB phosphorylation at Ser-133 and p-CREB strongly binds to the chromatin regions of CRE binding sites in the* MUC5B* promoter region, thereby inducing* MUC5B* gene expression in bronchial epithelial cells.

Inflammation is well known to be involved in the recurrent injury and repair in IPF. Therefore a better understanding of various inflammatory mediators that influence MUC5B overproduction in airway epithelial cells is potentially important for establishing a therapeutic strategy for treating MUC5B overproduction and IPF.

## 4. Conclusion

This review aimed to demonstrate the importance of* MUC5B* in the development of IPF and the potential mechanisms of* MUC5B* overexpression in airway epithelial cells. First, the localization of MUC5B in IPF lungs suggests a prominent role for injury and abnormal repair to the distal airway epithelium cells in IPF, inconsistent with the traditional view that IPF is a disease of the alveolar epithelium. It suggests that* MUC5B* overexpression in the distal airway may play a role in the development of IPF. Second, although there are limited data regarding the mechanisms of* MUC5B* overexpression in distal airway epithelium in IPF lungs, various studies have demonstrated that many factors modulate the expression of* MUC5B. *Besides the promoter variant rs35705950, which definitely causes an overexpression of* MUC5B* and appears to be predictive of IPF, some transcriptional factors, inflammatory mediators, and associated signaling pathways may also mediate* MUC5B* overexpression, providing clues for further research regarding the mechanisms of* MUC5B* overexpression in IPF, and may facilitate development of novel treatments for this fatal disease with limited treatment options.

## Abbreviations

IPF: Idiopathic pulmonary fibrosis
FIP: Familial interstitial pneumonia
TERT: Telomerase reverse transcriptase
TERC: Telomerase RNA component
PARN: Poly(A)-specific ribonuclease
RTEL: Regulator of telomere length elongation helicase
SPC: Surfactant protein C
SPA2: Surfactant protein A2
TOLLIP: Toll interacting protein
FOXA2: Forkhead box A2
AP-1: Cativator protein 1 transcription factor
LPS: Lipopolysaccharide
MAPK: Mitogen-activated protein kinase
ROS: Reactive oxygen species
EGFR: Epidermal growth factor receptor
TLR4: Toll-like receptor 4
TLR2: Toll-like receptor 2
PGD2: Prostaglandin D2
DP1: D-prostanoid receptor.
